# Sharpening the cutting edge: additional considerations for the UK debates on embryonic interventions for mitochondrial diseases

**DOI:** 10.1186/s40504-016-0046-2

**Published:** 2017-01-13

**Authors:** Erica Haimes, Ken Taylor

**Affiliations:** PEALS (Policy, Ethics and Life Sciences) Research Centre, Newcastle University, 4th Floor Claremont Bridge, Claremont Road, Newcastle upon Tyne, NE1 7RU UK

**Keywords:** Mitochondrial diseases, Three-genome embryo, UK regulation, UK debates, PNT/MST, Mitochondrial donation

## Abstract

In October 2015 the UK enacted legislation to permit the clinical use of two cutting edge germline-altering, IVF-based embryonic techniques: pronuclear transfer and maternal spindle transfer (PNT and MST). The aim is to use these techniques to prevent the maternal transmission of serious mitochondrial diseases. Major claims have been made about the quality of the debates that preceded this legislation and the significance of those debates for UK decision-making on other biotechnologies, as well as for other countries considering similar legislation. In this article we conduct a systematic analysis of those UK debates and suggest that claims about their quality are over-stated. We identify, and analyse in detail, ten areas where greater clarity, depth and nuance would have produced sharper understandings of the contributions, limitations and wider social impacts of these mitochondrial interventions. We explore the implications of these additional considerations for (i) the protection of all parties involved, should the techniques transfer to clinical applications; (ii) the legitimacy of focussing on short-term gains for individuals over public health considerations, and (iii) the maintenance and improvement of public trust in medical biotechnologies. We conclude that a more measured evaluation of the content and quality of the UK debates is important and timely: such a critique provides a clearer understanding of the possible, but specific, contributions of these interventions, both in the UK and elsewhere; also, these additional insights can now inform the emerging processes of implementation, regulation and practice of mitochondrial interventions.

## Introduction

In October 2015 the UK became the first country to enact legislation, originally agreed by Parliament in February 2015, to permit the clinical use of embryos that have been constructed using genetic material from three people. The purpose of constructing such embryos is to allow women who carry certain types of incurable mitochondrial disease to attempt to have genetically related children who will, it is hoped, be free from such diseases. Following a decade of research, two IVF-based techniques have been developed: (i) pronuclear transfer (PNT) and (ii) maternal spindle transfer (MST), both often erroneously referred to as ‘mitochondrial donation’ (Haimes and Taylor 2015).^1^ In both techniques the intending parents’ DNA is transferred into an egg (containing healthy mitochondria) provided by another woman, from which the nuclear DNA has been removed. Both techniques therefore result in a ‘constructed’ embryo that contains DNA from three people: nuclear DNA from the intending mother, nuclear DNA from the intending father and mitochondrial DNA from the woman acting as the egg provider. Elsewhere we have described the resulting embryo as a ‘three-genome embryo’ in a challenge to the more commonly used phrase of ‘three parent babies’ (Haimes and Taylor 2015).

Major claims have been made about the quality of the UK debates leading up to the February 2015 decision and the significance of those debates for UK decision making on other biotechnologies, and for other countries considering whether to approve mitochondrial therapies. For example, the UK’s Government Chief Scientific Adviser, Sir Mark Walport, asserted that ‘approval in the United Kingdom of mitochondrial donation provides a blueprint for future decisions on modifying the genome’ (Hawkes 2015). In December 2015 Walport also said of the UK, ‘…we are good at the science, we’re very good at the regulation and we’re very good at the public discussion.’ (Knapton 2015).

However, in this article we suggest that these claims about the quality of the UK debates around mitochondrial interventions are somewhat over-stated. Through a systematic analysis of the published documents and statements that were produced by key participants in those debates we identify ten areas where greater clarity, depth and nuance would have produced a more rounded debate over the field of mitochondrial interventions. We suggest that a more careful consideration of these areas sharpens understandings of the contributions, and limitations, of mitochondrial interventions as well as of their wider socio-ethical impacts. Our analysis of the ways in which wider debates over mitochondrial interventions could be improved is very timely. In December 2016 the UK regulatory agency, the Human Fertilisation and Embryology Authority (HFEA) will decide whether to issue licences to clinics to allow them to pursue these clinical applications; also, other countries, such as the USA, are considering whether to approve such interventions. Our review of, and suggestions for improving, the UK deliberations, contribute to discussions over mitochondrial interventions in other jurisdictions; it also contributes to a growing literature on innovations and governance in biotechnologies more generally.

## Background

Mitochondrial interventions, hereafter referred to as PNT/MST to better reflect the actual techniques under scrutiny, have provoked much discussion because they involve changing the genetic inheritance of any children born, in novel, untried, irreversible ways that could never occur through ‘natural’ conception. Further, this genetic change will be perpetuated in future generations who are descended from any girls born using this approach, because it is a form of germline modification. As noted above, the use of these techniques is justified by the aim to prevent the maternal transmission of some types of mitochondrial disease. It is therefore pertinent to provide some contextualising information (see also Bredenoord and Braude 2011).

### Mitochondria and mitochondrial diseases

Mitochondria are small structures (organelles), multiple copies of which are found in almost all human cells,^2^ including gametes; they are the sites at which the energy that sustains life is produced. Uniquely amongst organelles, mitochondria possess their own DNA (mt-DNA) which is distinct from the nuclear DNA that contains the majority of a person’s genetic material. The mitochondrial genome is composed of 37 genes, 13 of which code for proteins involved in energy production. It is estimated that there are around 21,000 genes in the nuclear DNA (Pennisi 2012).

Mitochondrial diseases, which result from a reduction in the capability of mitochondria to produce energy, are complex. There are many different mitochondrial diseases, which range in severity and age of onset, some of which are difficult to diagnose. Both sexes can suffer from mitochondrial diseases and the same disease can affect different individuals to varying degrees. Some mitochondrial diseases are caused by mutations in the mt-DNA, though not every mitochondrion may carry the pathogenic mutation; it is this variation in ‘mutation load’ that accounts for the variability in the way the mitochondrial diseases affect different individuals.

### Inheritance

Mitochondrial-DNA is inherited only through the female line, as all the mitochondria in a developing embryo are derived from those originally present in the egg. Complex and incompletely understood patterns of replication of mitochondria in the developing embryo mean that it is rarely possible to predict accurately the severity of mitochondrial diseases. Nonetheless, if a woman has a disease-causing mt-DNA mutation, she will inevitably pass that mutation on to all her children. It is maternally transmitted mt-DNA disease, as opposed to mitochondria diseases from *de novo* mutations, that clinician-scientists^3^ hope to eradicate from families through the use of PNT/MST.

### Regulation

All research and treatment in the UK that uses gametes and embryos is regulated by the Human Fertilisation and Embryology Authority (HFEA), the body established by the Human Fertilisation and Embryology (HFE) Act (1990). The original HFE Act has been amended on several occasions to take account of developments in science; each amendment requires debate and a majority vote in Parliament to be enacted. In 2001 an amendment was made to permit the HFEA to license research that would increase ‘knowledge about serious diseases’ and to enable ‘any such knowledge to be applied in developing treatments for serious disease’ (HM Government 2001). In 2005 it was argued that mitochondrial diseases were ‘serious’ and the HFEA granted a research licence to clinician-scientists at Newcastle University^4^ to permit them to conduct research on PNT/MST, though at that point it remained illegal to use an embryo created by the techniques for clinical applications. Then in 2008 the HFE Act was amended,

… introducing new powers which allow for Regulations to be passed by Parliament that will allow techniques that alter the DNA of an egg or embryo to be used in assisted conception, to prevent the transmission of serious mitochondrial disease (HFEA 2013, 9).

In May 2010 the HFEA reviewed developments in the science and, in collaboration with the Newcastle clinician-scientists, made a request to the Government to invoke the provision for such Regulations to be debated. This led to debates in Parliament and the subsequent change in the law, in February 2015. This change was then enacted on October 29^th^ 2015.

## Sharpening the UK debates on the clinical applications of PNT and MST

In analysing the UK debates we conducted a systematic review of the major regulatory, scientific, clinical and bioethics contributions in the public domain that preceded the February 2015 decision. These included: (i) all Parliamentary debates from both the House of Commons and the House of Lords; (ii) all reports from the HFEA relating to mitochondrial interventions, including its public consultation on this subject; (iii) major bioethics commentaries, including the 2012 report from the Nuffield Council on Bioethics on ‘Novel techniques for the prevention of mitochondrial DNA disorders: an ethical review’, and (iv) contributions from patient groups. We also reviewed how these discussions were represented in the mass media and have used elements of these to illustrate how arguments made in one sector became translated across to other sectors (for example, from the scientific to the mass media to the Parliamentary debates: see section (v) below^5^). An analysis of these debates reveals a number of areas that were neglected or marginalised or that lacked depth and nuance in certain key aspects despite being crucial to the development and implementation of these techniques. We have identified ten areas that would benefit from greater consideration and suggest ways in which that further scrutiny might develop. We then discuss the implications of these issues for ongoing debate on treatments for mitochondrial diseases and for the governance of other biotechnologies in the near future, in the UK.

(i) Other ways to have a baby.

One of the most prominent gaps in the UK debates of mitochondrial interventions was the absence of consideration of other reproductive possibilities available to affected women, accompanied by a failure to weigh, systematically, the costs and benefits of each option against the others. Instead the new science of mitochondrial intervention was the starting point for deliberation and was presented in some quarters as the only desirable way in which women with faulty mitochondria could have healthy babies. The alternatives of ‘traditional’ full egg donation,^6^ surrogacy with egg donation, or adoption, were rarely discussed, let alone given detailed or informed consideration. For example, even an organisation as well versed in assisted conception as the HFEA made no reference, in its advice to Government resulting from its public consultation, to these other ways that affected women could have a healthy child. This suggests that these alternatives were not prominent in their deliberations or in the consultation processes (HFEA 2013).

Similarly, Chinnery et al. (2014) state,

Mitochondrial DNA diseases are transmitted maternally, and for families carrying these mutations, a major, and justifiable, desire is to have unaffected children. For some women, pre-implantation or prenatal diagnosis may be helpful [4,5], but for other women, these techniques will not result in disease free offspring and the only options available are either oocyte donation or mitochondrial replacement at the oocyte or zygote stage. The need for this technique for these families is well established, as are the experimental methods that are required for mitochondrial replacement [6–8] (2014, 1, emphasis added).

In claiming that the ‘need’ for mitochondrial interventions is ‘well-established’, Chinnery et al. (2014) provide no evidence from affected women or families^7^ themselves. However, Herbrand (2016) suggests that such women face much more complex reproductive choices and do not regard PNT or MST as the simple solutions that they were presented as being.^8^

(ii) Valorising ‘genetic connections’.

The difference between the above-mentioned alternatives and PNT/MST is that, with the latter, the person conceived would be genetically related to the intending parents. The observation that PNT/MST provided the only route through which an affected woman could have genetically related children was the explicit starting point for the Parliamentary debates in 2015. The House of Commons debate opened with the statement,

Ellison: … The techniques provided for by these regulations offer the only hope for some women who carry the disease to have healthy, genetically related children who will not suffer from the devastating and often fatal consequences of serious mitochondrial disease (Hansard, 2015a: Col 160).

Similarly in the House of Lords, The Parliamentary Under-Secretary of State at the Department of Health opened the debate saying,

My Lords, the purpose of the regulations is to enable women to have their own genetic children, free of terrible disease caused by disorders in their mitochondrial DNA (Hansard, 2015b: Col 1569).

Such opening statements set the terms for these debates and thereby limited the opportunity to debate alternative approaches, by establishing the view that genetic relatedness was a key consideration driving the development of the techniques.

Sometimes the centrality of concerns around genetic relatedness was simply assumed, particularly in the media coverage where this was often coupled with another trope in these discussions, a reference to the plight of named individuals and families. McKie, for example, noted in The Observer newspaper, that one woman whose son died of a mitochondrial disease has a surviving daughter and wants,

this new technique to be given the go-ahead, so my daughter will have healthy mitochondria and can have children who will not die when they are teenagers, as her brother Adam did (2014, 19).

A Daily Mail article reported that ‘Women carrying damaged mitochondria can also miscarry repeatedly and often face the heartbreaking choice of whether it would be best to remain childless’ (Macrae 2014). Neither of these pieces (typical of the many published) mention the alternative routes that affected women could take to become mothers of healthy children, again reinforcing the assumed importance of the genetic connection sustained by PNT/MST. The Macrae article also raised the notion of ‘choice’ which was common in many contributions to these discussions. When questioned on the possibility of using non-PNT/MST methods to conceive, an affected woman interviewed on a BBC national news programme on the day of the House of Commons debate, said that the mitochondrial techniques would give her, and women like her, ‘choice’. She did not expand on that though the insinuation within the context of the discussion was that this is the choice to have a genetically related baby; this was not pursued by the interviewer, as if ‘choice’ in and of itself was an unquestioned value.

Whiteman (2013) argues,

‘Choice’ is a word that has, arguably, become near-ubiquitous in UK political discourse. It has an air of simplicity in meaning, ingrained in casual use and yet the word is value-ridden. The term ‘choice’ in the context of health care and beyond has become an extension of expressing (or choosing) preferences. To have choice implies that there is the opportunity for an individual to have what they want, when they want it; demand if you will (2013, 148).

This emergence of the language of choice and consumerism in the discourse around health care provision in the early 2000s coincided with the long period of development of PNT/MST (Fotaki, 2010, 898). Nonetheless, in the context of the radio interview and other discussions around mitochondrial interventions, the notion of ‘choice’ was used to *close down* consideration of existing alternatives and to *promote* the new interventions, by those who were advocates for the science and by those who prioritised genetic relatedness between parents and children. Genuine discussion of choice in conception and reproduction (and more generally) would clearly have been an important aspect to add to these debates, given that families in twenty-first century UK are composed of individuals with a diverse range of genetic relationships, or none at all.

However, it is important to note that the valorisation of ‘choice’ and ‘genetic connections’ in this context was heavily skewed towards the assumed interests of the intending parents. Any ‘child’ resulting from these interventions will not be allowed access to identifying information about the woman who provided the egg and who therefore made that child’s existence possible. A person conceived through PNT/MST will be able to apply for information to confirm that s/he is the result of ‘mitochondrial donation’ and also be given information about the egg provider’s personal and family medical history, the screening she underwent prior to egg provision, and any other information lodged by the egg provider. However, the person conceived cannot be given ‘any information which may identify the mitochondrial donor’ (HM Government 2015). The absence of discussion about the longer-term interests of the person conceived is perhaps attributable to the fact that s/he was generally referred to, and portrayed in all the discussions, as either ‘a baby’ or ‘a child’, with little or no acknowledgement of the fact that s/he will become a fully independent adult with his/her own views about the importance, or otherwise, of ‘choice’ or ‘genetic connections’^9^ (Haimes 1998). Crouch (2016) has similarly commented that the UK discussions tended to focus on the needs of the intending parents rather than those of the person conceived, either as a child or in the longer term.

(iii) Safety issues.

As fundamental as the question, ‘what alternatives are available?’ is the question, ‘how safe is the proposed new intervention?’ Questions of the safety of PNT/MST were addressed by a Safety Review Panel (hereafter, the Panel) convened by the HFEA, to ‘review the latest evidence of safety and efficacy’ (HFEA 2014, 9).

The Panel’s third report comments,

From a medical or scientific point of view all novel treatments pose essentially the same question: when is a treatment safe to offer? Research can never answer every question before a new treatment is offered, nor can it be expected to guarantee safety or efficacy when applied for the first time in the clinic. It can only serve to reduce the risk and to highlight areas that need close attention. … the panel concluded both in 2011 and 2013 that the evidence available at those times did not suggest that the techniques are unsafe. The direction of travel remains the same, and the panel therefore come to the same conclusion in this report (HFEA 2014, 5, emphasis added).

Clearly the argument that it cannot be fully known whether new treatments are safe until they are tried in human beings is correct but it is questionable whether the appropriate comparator was addressed by the Panel. Medicines or medical devices that do not behave as safely as expected might well affect the first individuals to receive them, but PNT/MST are interventions of a different order, with the potential to affect the whole human species, rather than a series of individuals, because they change the germline. Given that there are existing alternative ways for affected women to try to conceive, it can be argued that a stronger indication of safety should have been required for the introduction of the new techniques.

This suggestion is supported by the number of scientists who questioned claims about the safety of PNT/MST during the period of the UK debates. Ishii (2014) raised concerns about: (i) the lack of specific knowledge of the link between particular genetic mutations and ‘dysfunction at cellular organ and systematic levels’ (2014, 153); (ii) the potential for problems to arise from mt-DNA and nuclear DNA interactions, and (iii) the unknown effects on epigenetic programming during development of any embryo resulting from PNT/MST. Ishii was also concerned that other countries might follow the UK lead before the safety questions had been adequately addressed. The stem cell scientist Paul Knoepfler (2014) raised similar concerns and additionally identified the potential for ‘preferential replication’ of any damaged mitochondria carried over to the constructed embryo from the affected woman’s egg; this would result in increasing numbers of damaged mitochondria in the developing embryo at the cost of healthy mitochondria, so the person conceived would still inherit the mother’s pathogenic mitochondrial DNA. Morrow (2014) raised concerns about potential problems from mismatching nuclear and mitochondrial DNA and also pointed out that it is,

a contradiction to claim [as some proponents do: see section (vi) below] that mtDNA is not important for an individual's characteristics … while at the same time acknowledging that changes in the mitochondrial genetic code are important for an individual's risk of disease (2014).

St John (2014) identified the need to avoid any transfer of damaged mitochondria from the affected woman’s egg, and the practical difficulty of doing so, describing animal experiments that have shown the persistence of damaged mitochondria. He also raised concerns about epigenetic effects and mismatching of nuclear and mitochondrial DNA.

A prominent theme in discussions about safety was whether it is possible to separate ‘safety’ issues from ‘ethical’ issues. The Panel set explicit boundaries around their contribution to the debates by saying, ‘this review focuses exclusively on the science and the safety and effectiveness of these techniques; it does not consider the ethical and legal issues that are raised by such techniques…’ (HFEA 2014, p9). Similarly, Walport was reported to say:

People have extreme beliefs about whether it is right for humans to tamper with embryos in any way at all. Sometimes the values discussion gets conflated with the science discussion. We shouldn't pretend we're having an argument about science when we're having an argument about values. People need to say [why] they really object and not duck why they object by pretending there is something wrong with the science (Knapton 2015).

Nonetheless, some Parliamentarians echoed the above safety concerns while making clear that they had principled, ethical, objections, distinct from, but additional to, concerns about safety. For example, Bruce stated,

I want to speak against the Government motion… Human mitochondrial disease is a dreadful condition and, as a caring society, we must do all we can to address it… in an ethical manner and with proper regard for safety. I believe that the regulations we are considering today fail on both counts—ethics and safety—and that they are inextricably interlinked. Let me be straightforward: I do oppose these proposals in principle. However, that should not prevent my concerns regarding their safety from being given a fair hearing (Hansard 2015a: col 168).

Equally, neatly separating safety from ethical issues was not easy for the participants in the HFEA consultation:

The public dialogue and consultation work we undertook was focused on gathering and understanding public views on the social and ethical issues associated with mitochondria replacement. We wanted to explore their views independent of any questions of safety and efficacy. In practice, however, people’s views on these issues tended to be linked to questions of safety; this was a strong theme through all the responses. Sometimes, safety concerns become a proxy for concerns about ethical and social issues, which are often hard to express. On other occasions, support for mitochondria replacement dipped when the scientific evidence was less clear (HFEA 2013, para 6.8, emphasis added).

Clearly, issues about the safety of any intervention cannot be neatly distinguished from socio-ethical concerns, not least because it can never be morally correct to offer interventions that are known to be unsafe, except under exceptional circumstances and where no alternatives exist. On the more general point, there is also not as clear a divide between science and values as some contributors suggested: as we have seen above, PNT/MST are partly being offered in the first place because of the largely unstated valorisation in the scientific literature of genetic ties between parents and children. The vast literature from Science and Technology Studies identifies the numerous ways in which ‘science’, far from being an external source of objective knowledge, is inextricably shaped by, and shapes, wider social values, institutions and processes (e.g. Jasanoff 2004).

It is worth noting that papers published since the 2015 UK legislation indicate that earlier concerns about the safety of PNT/MST are justified. Callaway (2016) reports that work carried out by Egli’s group in New York (Yamada et al. 2016) found, as Knoepfler feared, that small amounts of the intending mother’s damaged mt-DNA might be carried over and ‘outcompete healthy mitochondria in a child’s cells and potentially cause the disease [that] the therapy was designed to avoid’. Further evidence of this phenomenon was reported in Nature in November 2016 (Kang et al. 2016; Cook 2016b). Egli argued that this problem would ‘defeat the purpose of doing mitochondrial replacement’ and recommended that the procedure not be used in the meantime, arguing ‘I don’t think it would be a wise decision to go forward with this uncertainty’ (quoted in Callaway, 2016).

This was followed by a letter to Nature from the Newcastle team reporting the first pre-clinical studies on PNT and acknowledging the importance of reducing mt-DNA carry over. The authors concluded that ‘PNT has the potential to reduce the risk of mtDNA diseases, but it may not guarantee prevention’ (Hyslop et al. 2016, 2) and that therefore ‘it should be considered in combination with prenatal screening’ (2016, 4). The lead Newcastle scientist, Professor Sir Doug Turnbull, was reported by the BBC as saying their letter sounded ‘a note of caution’. Surprisingly, however, the BBC headlined their article with the words: ‘Three-person babies IVF technique “safe”’ (BBC 2016). The Times newspaper assumed a smooth and unruffled progression to successful implementation, headlining a piece that reported the Newcastle letter with the words: ‘Three-parent babies could be born next year if new IVF wins approval’ (Moody, 2016). Rather more cautiously Knoepfler was quoted as saying that both the Yamada paper and the Newcastle letter ‘clearly indicate that the field is not ready to use this technology to create actual people. It would be reckless to do so… To their credit the [Newcastle] group acknowledges this challenge…’. Knoepfler goes on to say that ‘the legislative approval of this technology for use in humans in the UK last year, based in part on vigorous claims from proponents in the UK that there were plenty of data already, was more political than scientific.’ (Cook 2016a).

(iv) The focus on children and severe forms of disease.

A facet of the discussion that made such a political decision possible was the clear focus on babies and children, and on the most severe forms of mitochondrial diseases, by the mass media and patient advocacy groups. This is rather misleading since many mitochondrial diseases only manifest themselves in adults and have varying degrees of severity (NCoB 2012, Dimond 2015). It would have been more transparent (and the calculations of the numbers of affected persons would be higher) if the case in favour of allowing these techniques had reflected the full range of affected persons.

It could be argued that the devastating conditions that affect babies and young children are predominantly the conditions that the proposed techniques will be used to prevent. However, the women who are the targeted beneficiaries of PNT/MST have mitochondrial disease themselves and yet have a quality of life that has enabled them to get to the point of wanting to start a family; this is an indication that the impact of mitochondrial diseases is much more variable than presented in the debates. Therefore the focus on babies and children was a missed opportunity to raise awareness of the full range of mitochondrial diseases, and their manifestations, and of the differing experiences of living with such conditions.

However, that focus also had the effect of suggesting that affected babies would be the beneficiaries of the proposed intervention, which is clearly not the case. A healthy child might be the *result* of the intervention, but children affected by mitochondrial disease are not the *site* of the proposed intervention.

(v) The potential for raising unrealistic expectations.

As we have said, not all forms of mitochondrial disease are caused by mutations in the mt-DNA, but it is only disease caused by such mutations that the clinician-scientists hope to tackle using PNT/MST. When developments on PNT/MST were reported in the media, however, the complexities and nuances of the nature of mitochondrial diseases were often lost, leaving readers with the impression that ‘mitochondrial diseases’ can be ‘prevented’ or ‘eliminated’. For example, Collins (2012) in the Daily Telegraph quoted Professor Turnbull as listing specific examples of mitochondrial disease, including muscular dystrophy and ataxia and stating that the hope for PNT/MST was to ‘totally prevent the transmission of these diseases’ (Collins 2012). The journalist, however, then went on to cite the number of children (around 200) estimated to be born each year with *any* form of mitochondrial disease and did not clarify that only a smaller proportion of these had the conditions Turnbull described. Another example was a Guardian piece by their science editor, Ian Sample. Titled ‘”Three-parent babies” cure for illness raises ethical fear’, the article was not primarily concerned with ethical fears, but rather was a wide-ranging description of the potential of the work being done on PNT/MST. The article showed the same potential for confusing readers by referring to ‘mitochondrial disease’ as if this only arises from mt-DNA mutation, and gave the impression that all women affected by ‘mitochondrial disease’ would benefit from the new interventions. Sample also wrote that,

In Newcastle, Turnbull is working on ways to eliminate the risk of disease by replacing the mother's faulty mitochondria wholesale with those from a healthy donor (2012).

Again, this does not reflect the complexity of the situation, a position that was exacerbated when he quoted the leading American clinician-scientist working on MST,

Mitalipov says funding restrictions mean he cannot take the research on in humans. “We hope the UK takes it further. We have a way to prevent transmission of these diseases in children. It has to be tested or we will never know if it works,” he says (Sample 2012).

The lack of clarity about the range of conditions that will be valid targets for attempts to prevent transmission from mother to child is evident; the overall impression is that all mitochondrial diseases will be prevented.

Such lack of clarity might be dismissed as merely typical of journalistic oversimplification, were it not for the observation that some policymakers appeared to be susceptible to the misconceptions that can arise from these stories. The Member of Parliament Luciana Berger began her contribution to the House of Commons debate on legalising PNT/MST for clinical use by noting that she had followed the ‘public debate’ for ‘recent weeks and months’, and attended a Parliamentary event where ‘we heard representations from both sides’. She reached the conclusion that,

We have within our reach the possibility of eradicating mitochondrial disease from families who have been blighted by it for generations: families who have endured a disease for which there is no cure, who have suffered daily battles with painfully debilitating symptoms, and who have sadly lost their children prematurely (Hansard 2015a: Column 164).

However, as Herbrand (2016) highlighted, a later exchange in the debate revealed a fundamental misunderstanding,

Gillan: Can [you] confirm that mitochondrial disease from the nuclear DNA will remain in our population even after this treatment is licenced?

Berger: […] it is not something I have been made aware of, and it certainly has not come up in any of the discussions or debates that I have attended (Hansard 2015a: Column 168).

The conflation of ‘mitochondrial disease’ with specific serious mitochondrial diseases caused by mt-DNA mutations, plus the lack of clarity that ensues from the conflation of ‘preventing transmission’ with the numbers of children estimated to be affected by *all* mitochondrial diseases, results in the inflation of hope and expectation for the proposed interventions; not least expectations about the public health impact of permitting PNT/MST.

This sort of discourse in the UK debates meant that PNT/MST were not systematically evaluated against other necessary interventions for the wider range of mitochondrial diseases; it also meant that the possibility of *new* mt-DNA mutations resulting in mitochondrial diseases featured little in these discussions.

(vi) The paradox of small numbers…or the insignificance of significant genes.

As noted above the human mitochondrial genome is very small in comparison to the total size of the human genome. This fact was deployed in interesting ways by some proponents of PNT/MST. For example, the UK’s Chief Medical Officer, Dame Sally Davies, said:

It is important to remember that mitochondrial DNA represents less than 0.054% of the total DNA, and is not part of the nuclear DNA, which determines our personal characteristics and traits such as personality, hair and eye colour. (Davies 2015).

Similarly, a campaigning patient group, the Lily Foundation, which provides help and advice for parents with children who have mitochondrial disease, explained in some web-based information:

The baby would have <0.1% of their total DNA from a 3^rd^ person and this would be the mitochondrial DNA (mtDNA) in the donated mitochondria which enables normal energy production.

The overwhelming majority of the DNA, 99.9% (all the nuclear DNA that determines human characteristics) would only come from the mother and father (Lily Foundation 2015).

Parliamentarians echoed these statements; for example in the House of Commons debate on February 3^rd^, 2015,

McInnes (Heywood and Middleton) (Lab): As we have heard, mitochondrial DNA makes up a tiny proportion of our total DNA. … There are 37 genes in mitochondrial DNA, which is less than 0.01% 10 of our total DNA (Hansard 2015a: Col 179).

The Parliamentary Under-Secretary of State for the Department of Health, when opening the Lords debate, said ‘the DNA from the donor egg amounts to less than 1% of the resulting embryo’s genes’ and that ‘One important point to emphasise here is that none of the nuclear DNA, which determines our personal characteristics and traits, is altered by mitochondrial donation.’ (Hansard 2015b: Col 1569). Other examples abound. This is not the place to enter into argument with the claim that nuclear DNA ‘determines’ personality, other than to note that the presence or absence of mitochondrial disease is likely to have a profound impact on an individual’s personality (NCoB 2012; Baylis 2013; Haimes and Taylor 2015). More important to note for our analysis here is the way in which claims about the tiny portions of donated mt-DNA are configured. The implication of this repeated refrain is that, because the numbers being discussed are so small, the genetic material to which they refer is unimportant and insufficient to provoke concern. And yet, without these tiny amounts of mt-DNA, an affected woman would have no hope of bearing a healthy, genetically-related child – which of course is the whole point of these discussions in the first place. Minimising the contribution of the mitochondrial genome in numerical terms means that the proponents of the techniques were making paradoxical claims: saying these interventions and therefore these genes are both crucial (to prevent transmission of mitochondrial diseases) but are also essentially insignificant because they are only few in number. The insinuation is that opposing an intervention which involves such a small amount of genetic material could be nothing but unreasonable.

A further effect of establishing the notion that the mitochondria from the ‘healthy egg donor’ are providing only a small number of genes, is that it diminishes the importance of, and indeed helps to render invisible, the women who provide those ‘healthy’ mitochondria, and the labour involved in providing them, as we discuss in section (ix) below.

(vii) The number of affected individuals and families.

Just as numerical calculations were deployed to minimise and deflect concerns about the proposed interventions, they were also deployed to strengthen the case in favour of these interventions, albeit in confusing and inconsistent ways.

In a letter to Nature in 2010, the Newcastle group reported that,

[m]utations in mitochondrial DNA (mtDNA) are a common cause of genetic disease. Pathogenic mutations in mtDNA are detected in approximately 1 in 250 live births and at least 1 in 10,000 adults in the UK are affected by mtDNA disease (Craven et al. 2010, 82).

Using publicly available information, we can use these figures to estimate that around 5280 adults in the UK are affected by mt-DNA diseases.^11^ However, until 2015, the number of families to be treated through PNT/MST per year was often cited in debates as ‘around 10’ (for example (BBC 2013), Sample (2013)) though it is unclear from whence this figure originated. Just before the 2015 House of Commons debate, the Newcastle Group estimated that the ‘average number of births per year among women at risk for transmitting mtDNA disease’ was 152, from a population of 2473 affected women in the UK suggesting that in the future about 150 births per year could potentially benefit ‘if all women opted for the procedure’ (Gorman et al. 2015, 886-7). Again, using publicly available figures and an alternative approach to estimating the number of women who might be able to benefit from PNT/MST, it is possible to estimate that the number of children born each year who may be affected by serious mitochondrial disease is between 112 and 148.^12^ (See also Herbrand 2016).

Clearly the figures cited depend on the starting point for any particular calculation. Some of the confusion in the early discussions about the numbers of women, babies and families involved might have arisen from a lack of clarity about: (i) the number of women carrying faulty mitochondria; (ii) the number of those who might seek to get pregnant each year; (iii) the number of babies likely to be born from those women; (iv) the number of people in families likely to be affected by the birth of a severely affected baby; (v) the numbers of babies that could be conceived through natural pregnancy (and who would therefore be highly likely to be affected); (vi) the number of babies that could be conceived using IVF, knowing that IVF only has a success rate of about 30% (an often unacknowledged aspect in the ‘public discussion’). It is important therefore that the basis of any calculation is clearly established before making any claims or drawing any conclusions from those figures. The Newcastle Group clarified many of these issues through analysis of their patient registry and Office of National Statistics data (Gorman et al. 2015); it is to their credit that they published these findings ahead of the Parliamentary debates.

(viii) Regulatory oversight and capacity.

This lack of consistency and clarity was significant for the UK debates in another way. A requirement of the UK legislation was that the HFEA must review each application to use PNT/MST in a woman’s treatment, on a case-by-case basis; this step was consistently noted to be an important safeguard in the clinical application of these techniques. For example, an HFEA press release, following the passage of the regulations through Parliament, noted,

Each application will be decided on a case by case basis and in accordance with the latest scientific advice. An HFEA committee will determine whether individual patients and families have a particular risk of an abnormality in their mitochondrial DNA; and whether there is a significant risk that a child born with that abnormality will have, or will develop, a serious physical or mental disability, a serious illness or another serious medical condition (HFEA 2015).

While 10 cases per year might be manageable, the question of how the HFEA would deal with up to 150 cases per year was not part of the wider discussions. When asked a question^13^ about the possibility of 150 cases coming forward per year, the Chair of the HFEA echoed the claim that only around 10 applications were expected per year and that the Authority had the resources to cope with that number. No acknowledgement was made that the higher figure might be possible. It is a moot point as to what impact the higher numbers had on the views of Parliamentarians, but clearly the higher figures could be deployed to make the case that PNT/MST interventions are much needed, while the lower number was deployed to reassure all parties that regulatory oversight could and would be provided. Whatever the baseline figures, the work for the HFEA may be increased given the recent recommendation from the Newcastle Group that PNT should only be used in conjunction with embryo screening (Hyslop et al. 2016).

The variations in calculations of the numbers of cases, plus the failure to systematically consider the possible implications of the higher numbers for effective regulatory oversight, erodes confidence in Walport’s claim, cited earlier, that the UK are ‘very good at the regulation and… very good at the public discussion’.

(ix) The egg providers.

As we have detailed elsewhere (Haimes and Taylor 2015) the discussion of the egg providers (for both research and treatment), and the terminology used to describe them in the debates, was problematic. Little, if anything, was said about the role and contributions made by these women. Indeed it appears that their role was deliberately minimised: for example, the NCoB argued they should not ‘be given the same status in all aspects of regulation as a reproductive egg or embryo donor’ (2012, para 5.3) and the HFEA ‘Advice to Government’ reduced their status to that of mere ‘tissue donors’ (2013, para 1.13). The commitment of time, energy and generosity required from the egg providers and the invasiveness of the procedure through which the eggs are produced and collected for use in research or treatment were all but ignored. (For further discussion see Baylis 2013, Haimes 2013, Haimes and Taylor 2013).

This is surprising given the very simple fact that without the egg providers no research, let alone PNT/MST clinical interventions, would have been possible. However, this airbrushing^14^ (Haimes and Taylor 2015) which renders them invisible can be more clearly understood in light of other aspects of the UK debates, such as the playing down of the genetic contribution that egg providers will make to any offspring conceived (see section vi above), the unwillingness to consider other means of conception (see section i above) and the valorisation of the genetic connection between the intending parents and the person conceived (see section ii above). These reflect the longstanding uneasiness amongst clinicians and regulators about the role of third party conceptions (Haimes 1998, Turkmendag 2016), of which this is the latest manifestation. It is as though the egg provider is ‘the other woman’ whose necessary role in the process is all the more embarrassing precisely because it is necessary. While careful consideration does need to be given to the nature of the longer-term relationship between egg providers and those conceived, this is not achieved by disguising the role of egg providers but rather by acknowledging, and indeed celebrating, their vital contributions to these developments (Haimes and Taylor 2015).

(x) Rapid legislation:

A recurring theme throughout the UK discussions was that time was of the essence in changing the law to permit the clinical use of PNT/MST. For example in the House of Lords debate on 24/02/15,

Viscount Ridley (Con): … Finally, is it rushed? Far from being hurried, it has been under development for more than 30 years, under debate for 15 and under scrutiny for five. There is nothing slippery about this slope. There has been no rush. Now, however, that we have reached this stage there jolly well should be some reasonable haste on behalf of the women whose reproductive life is running out and who desperately want their own child, people such as Claire Wright, who is now 40 and who had to watch her son Jacob lose his smile on the way to a cruel death. Yes, there is understandable urgency. We would have to have very good reasons to argue that the ethical thing to do is to prolong her suffering and that of others like her. I cannot see those reasons (Hansard 2015b: Col 1588).

This need for rapid legislation was challenged on the grounds that the safety issues had not yet been resolved. Bruce, speaking in the House of Commons in September 2014, noted,

Bruce (Congleton) (Con): … Sir John Tooke, president of the Academy of Medical Sciences has said: “*Introducing regulations now will ensure that there is no avoidable delay in these treatments reaching affected families once there is sufficient evidence of safety and efficacy*.” In other words, Parliament should vote blind and sign off legislation permitting these procedures before the recommended experiments—some of them critical, regarding safety—have been completed (Hansard 2014: Col 94).

While an acknowledged opponent of embryo research in general, Bruce here is expressing concerns that Parliament would (and did) vote on the legislation with incomplete understanding of the safety of PNT/MST and therefore with an incomplete understanding of the possible long-term consequences for the affected women and any offspring they may have through using these techniques.

Support for the view that there were grounds for delaying legislation comes from primate research in the USA. This was given little exposure during the wider UK debates even though the HFEA safety panel (HFEA 2014) did note that Mitalipov’s group in Oregon had four five-year old male Macaques, conceived using MST techniques:

The group is seeking to establish the fertility status of the Macaques by entering them into a breeding programme and more focussed studies looking at physiological impact will be conducted. There remains one female Macaque who is 2–3 years old… that has not yet reached sexual maturity (HFEA, 2014, paragraph 3.3.3).

This means that it would only have been two to three years before the health of any offspring from the female Macaque could be established and therefore any long term negative consequences detected. Some Parliamentarians, such as Bruce above, clearly thought that waiting until these (and other) safety experiments had been completed would have been prudent. Therefore, within the terms of existing and imminent knowledge within the field, it could be argued that there was an over-hasty move to legislation.

We can see in the contrast between Ridley and Bruce a tension, also unexplored in the wider discussions, between the very real needs of individuals (named individuals in particular) and consideration of the wider, public health, benefits, risks and costs to society as a whole of the introduction of these techniques. As Dawson argues, a focus on individuals reflects trends elsewhere, but at a cost:

Much contemporary medical ethics focuses on the values considered to be crucial in protecting the individual. Such values are important, but other values, more visible in public health practice and related to societies, populations and communities, are just as important (Dawson, 2015, p109).

Similarly, Petrini and Ricciardi (2015) describe the need for ‘the balancing of individual rights and collective interests, which are often in conflict’ (2015, 270). It could be argued that legalising PNT/MST operates in an inverse manner to public health measures such as vaccination, which requires that parents take a risk with their child for the benefit of the population (Wood-Harper 2005) since the former entails taking a risk with the future of the population, through the unknown and irreversible consequences of modifying the germline, in favour of the immediate, hoped-for benefits to individual families.^15^

## Discussion

In summary, we have identified ten areas in which the UK discussions of PNT/MST initiatives would have benefitted from greater clarity, depth and nuance; in each area we have highlighted information and knowledge that could have been more prominent and contributed to a more fully rounded debate. Through this analysis we have sharpened awareness of the wider social implications of this cutting-edge science, by contextualising these developments and drawing more centrally into consideration questions concerning, amongst other things: the reproductive choices available; the kinship relationships created; the relationship between safety and ethics; the characterisations of the assumed beneficiaries; the ambitions and limitations of the proposed interventions, and the challenges for regulatory capacity. These contributions are important since, while the UK legislation has already been changed, details of implementation, regulation and practice have not yet settled into place; alerting the field to the need to consider these areas, and the challenges they represent, is a timely and useful task. While some of these topics constitute a critique of the UK debates, others support the claims of those eager to translate the science into clinical applications as fully as possible; the point of this analysis is not simply to pick holes in existing debates but to assist in ensuring that the full range of deliberations is as strong and clear as possible. Together, the examination of these areas enhances our understandings of the contributions that mitochondrial interventions can make and enhances our understandings of how this new science can best be developed to benefit those who want and need it most; it also assists the achievement of a more measured evaluation of the wider social and ethical impacts of these developments.

This more moderate, and modest, assessment is necessary since the existing UK debates on mitochondrial interventions, including their influence on the legislative process, raise questions about the governance of this, and associated areas of biotechnology, in at least three related areas. **(i) The protection of women, offspring and patients.** The protection of research participants and of patients is one of the key roles of any governance system in biomedical sciences. In research involving assisted reproduction, the protection of any persons born and that of the providers of gametes or embryos must also be considered. However, in the case of the clinical applications of PNT/MST we have demonstrated that the regulations passed in Parliament, and which will be implemented by the HFEA, were influenced by, and so repeated, the weaknesses prevalent in the wider discussions. For example, the ‘strategy of persuasion’ (Haimes 2014) adopted by proponents of the techniques acted to reduce the egg provider to just a ‘tissue donor’ (HFEA 2013). This results in her being considered in law in a more instrumental way than other women who provide eggs for treatment in other circumstances, insofar as she is seen merely in terms of her provision of healthy mitochondria rather than in terms of her own agency in acting in such altruistic ways or for her wider contributions to the field as a whole (see section (ix) above and Haimes and Taylor 2015). Similarly, the protection of any people born as a result of PNT/MST should also require that the techniques used in their conception be as safe as possible, in accordance with agreed clinical standards (see section (iii) above). However, in confidently dismissing opponents’ questioning of safety as being rooted in questions of values, some proponents of the interventions (Jack 2016) closed down the possibility of open discussion that is a requirement of good systems of governance. This raises questions about the effective protection of the offspring and the women who bear them (Crouch 2016). There is no doubt that the clinician-scientists involved in developing PNT/MST have the best interests of their patients at heart and are concerned for the safety and wellbeing of all involved. However, the manner in which safety concerns, raised in good faith, were dismissed by some supporters of PNT/MST interventions is not what might be expected in a high quality discussion.

**(ii) The legitimacy of focussing on short-term benefit**. Given the concerns over safety and the other issues raised above, there are, as we have seen, valid questions over the legitimacy of the law being changed when, and how, it was. The case of PNT/MST shares similarities with earlier debates on biotechnology studied by Brown and Benyon-Jones (2012). In their work on UK policymaking and regulation of xenotransplantation and human-animal hybrid embryos these authors identified a process which they termed ‘reflex regulation’, defined as,

…a sustained and default assemblage of conditioned and habitual regulatory behaviours and impulses that adversely limit potentially more critical and reflexive regulatory deliberation (Brown and Benyon-Jones 2012, 224).

They concluded that,

…reflex regulation is characterised by a focus on promissory risk and an emphasis on avoiding the loss of opportunity over that of avoiding other hazards and dangers. … This resonates closely with what we have called the ‘technocratic reflex’, i.e. policy culture’s enduring deference to scientific stakeholders’ definitions of risk in spite of a widely purported policy ‘turn’ towards greater inclusivity in deliberation concerning technoscientific futures (2012, 237).

In the case of PNT/MST we have seen both the emphasis on pressing ahead with legislation to enable the clinician-scientists to offer treatment quickly to affected women, accompanied by the effective (even if unintentional) silencing of criticisms of the science. We have highlighted the absence of informed discussion of the risks to wider society and the focus instead on the risks to individuals from not being offered treatment, despite the supposedly ‘open’ approach to policymaking and governance espoused by Government (HM Government 2013).

One means of achieving the better regulation envisaged by Brown and Benyon-Jones could result from the adoption of the principle of ‘Responsible Research and Innovation’ (RRI) (Stilgoe et al. 2013). This approach to the conduct of science has recently been gaining importance, particularly at European level (EU 2016). Stilgoe et al. identify how, despite recent approaches that include consultation or engagement with a range of stakeholders, in a bid to identify wider concerns about the purposes of and motivation for research, current governance arrangements for science are still focussed on the narrower, formalised means of risk assessment (2013, 1569).

In an effort to develop a framework for ‘responsible research’ Stilgoe et al. avoid the approach taken by others (e.g. von Schomberg 2011) who see RRI as a means of pursuing particular values and desired outcomes, and instead focus on the processes of governance that encompass ‘the distinctive social and ethical stakes’ in technological developments (quoted in Stilgoe et al. 2013, 1577). It is just this sort of focus on the wider societal interests in PNT/MST that our ten areas add to the UK debates and any subsequent regulation processes.

We can see that Government policymaking on PNT/MST prioritised the ‘promotional narratives of near-term future benefits’ (Brown and Benyon-Jones, 2012, 237) in which the views of some stakeholders, particularly the proponents of the science, were privileged over others. Examples of this were provided by the lack of systematic consideration of alternative ways women might have an unaffected child and also by the Chief Medical Officer’s statement that,

[s]cientists have developed ground-breaking new procedures which could stop these diseases being passed on, bringing hope to many families seeking to prevent their future children inheriting them. It’s only right that we look to introduce this life-saving treatment as soon as we can (Department of Health 2013).

**(iii) The risk to public trust.** We have raised concerns about the protection of those who may be involved in research and clinical applications of PNT/MST and about the legitimacy of the rapid legislative process by which clinical applications have been enabled. Such concerns have implications for the trust that wider publics might or might not have in biosciences. By pressing ahead without a more detailed and more nuanced discussion that could have involved more diverse publics, more thoroughly, the proponents of PNT/MST risk jeopardising the trust in medical scientists currently held by those publics (Castell et al. 2014, Marris 2015). As Brown and Benyon-Jones showed, the promise of areas such as human-animal hybrid embryos for research and medicine has been left unfulfilled, as indeed is the case for much that was expected of human embryonic stem cell research. Should the proposed clinical application of PNT/MST be less than hoped for, or worse still, cause harm, then wider publics are even more likely to come to distrust both the therapeutic promise of other new technologies and those who make such promises.

One means of building or maintaining public trust in the biosciences is through the design and implementation of good governance structures; however, as Harmon et al. note, this is not straightforward:

The modern biosciences require governance frameworks that are capable of simultaneously managing risk, coping with uncertainty, combating ambivalence, and building trust, all the while encouraging the delivery of those instrumental outputs that we value/demand (better health, new technologies, commercial reward). This multi-dimensional task makes the design and delivery of good governance frameworks … extremely difficult (Harmon et al. 2013, 31).

In Harmon et al’s view, which mirrors those of Stilgoe et al. (2013) and Brown and Benyon-Jones (2012), approaches to the governance of bioscience have tended to be inadequate. One reason for such inadequacy is that a key element of good governance is an assessment of the risk, in the wider, socially-defined, understandings of that term, posed by a particular technology or process. Harmon et al’s review of the work in this field leads them to conclude that the scientific understanding of the risks of biosciences are incomplete and ‘risk articulations are tentative and sound assessments are not yet within our grasp’ (2013, 26). In such a situation, legislators face a dilemma: should the law be used to prohibit certain technologies, or promote them, given that risks are largely unknown? A precautionary approach may be taken, but Harmon et al. consider this to be a contributing factor to the ambivalence around bioscience observed in wider publics.

Many authors have suggested that biotechnology governance frameworks would benefit by involving publics in their development, which, it is argued, would also lead to increased trust in biosciences (e.g. Harmon et al. 2013, 27). Public consultations are mechanisms frequently employed to involve publics in the legislative process; this was, as we have shown, an approach taken in the legalisation of PNT/MST therapies. However, the timing of the HFEA consultation in 2012 (let alone its conduct) can be seen as a concern, since, as Harmon et al. note, one-off, ‘snapshot’ consultations are inadequate for capturing the nuances of opinion about rapidly developing fields such as PNT/MST; they recommend ongoing dialogues between publics and policymakers instead (2013,28). These authors see a positive role for public engagement when done thoughtfully and robustly, providing policy-makers with ongoing evidence, assessment of risks and understanding of values that could inform governance frameworks over time. However, this was not aimed for, let alone achieved, in the case of PNT/MST.

## Concluding comments

Other jurisdictions might appreciate the usefulness of a more fully rounded debate. As Bredenoord and Hyun (2015) argue, when discussing whether the USA should follow the UK’s lead in permitting MST/PNT, ‘The bold leap from bench to bedside needs sufficient preclinical evidence and careful, long-term interdisciplinary research, including more ethics research’ (2015, 976). They recommend that the USA should take a more cautious path and argue, ‘A cautionary approach means that a wide variety of concerns should be taken seriously – wider than safety and efficacy alone’; the need is to find a balance between ‘taking appropriate precautions and not unnecessarily hampering innovation’ (2015,976). The US National Academies of Science, Engineering and Medicine report on mitochondrial disease (National Academies of Sciences, Engineering, and Medicine 2016) describes these interventions as raising ‘a novel collection of ethical, social and policy issues’ (2016, xiii). The starting point for their deliberations was ‘parental motivation’ for using these techniques in light of ‘alternative approaches to creating families’ and the ‘primary value’ to be considered when balancing risks and benefits of further clinical investigations was ‘minimizing the risk of harm to the child born as a result’ (2016, xv). They concluded that it is ethically permissible to conduct clinical investigations of these interventions but with two major restrictions: that investigation should be limited to women at risk of transmitting a serious mitochondrial disease and that only male embryos are transferred for gestation, ‘to avoid introducing heritable genetic modification during initial clinical investigations’. (2016, xv). While this second restriction raises its own ethical challenges in creating ‘experimental offspring’ (Taylor and Haimes 2012, NCoB 2012, 80), the USA is clearly taking more time to reach a decision and appears unlikely to follow the same path as the UK.

As we have shown, many important areas were neglected in the UK debates and the claims about the quality of those debates are over-stated. It is impossible to say what the outcome of the debates would have been had these more nuanced discussions been included. What we can suggest is that by leading the argument in the way that they did, focussing on the emotive and profoundly distressing effects of mitochondrial disease on babies, children and their families, and by failing to address a range of other issues in detail, the proponents of the technology leave themselves open to challenge in the future should the techniques fail to have as powerful an impact on mitochondrial diseases as some parties to the debates clearly expect them to have, or prove to be unsafe, or lead to other (perhaps more commercial and exploitative^16^) uses being approved.

The analysis presented here provokes the question of where the UK stands on the global stage in relation to mitochondrial interventions. Clearly the scientific endeavour in the UK is at the cutting edge of progress in this field. However, given that the UK legislation is the first example of the permissibility of genetic modification of human beings, and given the more cautious approach recommended for, and being taken by, the USA and Australia (de Lacey 2016), it could be argued that the UK is precariously out on a regulatory limb,^17^ until the many aspects discussed in this article are more clearly understood.

## Footnotes

1 As we have explained elsewhere, the terms ‘mitochondrial donation’ and ‘mitochondria donor’ are ‘misleading as they imply a direction of travel for the mitochondria that is in fact the opposite of what will actually occur. In the therapeutic application of PNT, an egg from the intending mother will be fertilised using sperm from the intending father. At the same time, an egg provided by a volunteer will be fertilised by the intending father’s sperm and that fertilised egg will be enucleated. The intending parents’ nuclear DNA, in the form of two pronuclei from their own fertilised egg, will then be transplanted into the enucleated egg which then acts as a ‘host’ to the transplanted nuclear DNA. MST will similarly involve the transfer of the intending mother’s nuclear DNA into an enucleated egg from the egg provider; that egg will then be fertilised by the intending father’s sperm. In other words, the egg provider’s mitochondria, whether in research or treatment contexts, are not moved anywhere. Therefore, rather than phrases such as ‘mitochondria donation/transfer/replacement’, a more accurate term would be ‘nuclear DNA hosting’, for both PNT and MST.’ (Haimes and Taylor 2015, 364-5).

2 Throughout this paper we discuss mitochondria as related to human life processes and health, though of course mitochondria are present in the cells of all multicellular organisms.

3 We use this term to emphasise that those leading the research on PNT/MST also tend to have clinical roles in diagnosing and caring for people affected by mitochondrial disease.

4 The authors are employed at the same university as that to which the clinician-scientists engaged in mitochondrial research and the recruitment of egg providers are affiliated, and our study was funded by the same organisation (Wellcome Trust) that funds the scientific research. However, our study was independent. We were assisted by a Project Advisory Group comprised of senior academics from other universities to ensure analytical objectivity.

5 We do not claim to have conducted a full blown ‘media analysis’ as our aim is to identify themes cutting across the different contributions to the UK debates rather than to analyse how one particular type of contribution (the ‘mass media’) shaped the overall debates. Newspaper and other media articles were identified via the systematic collation of all reporting (favourable or otherwise) of the work on mitochondrial disease interventions by Newcastle University-based clinician-scientists carried out by the University’s Press Office between May 2010 and September 2016, augmented by our own online searches.

6 Of course from the egg provider’s point of view all such measures entail giving a whole egg; however the distinction we draw here is between ‘traditional’ egg provision that contributes 50% of the genetic material to any resultant offspring, the donated egg completely replacing the intending mother’s egg, compared to egg provision in PNT/MST which will result in a smaller amount of genetic material being contributed and combined with both the intending parents’ DNA.

7 While it is always difficult to prove the absence of any feature, further evidence of the lack of consideration of alternative routes to reproduction can be found in utterances such as that from the lead scientist, Professor Turnbull, on greeting the news in December 2016 that approval might be given for the go ahead of clinical applications for PNT/MST: ‘This gives women who have mitochondrial DNA mutations reproductive choice, and I am delighted for them.'. The implication here is that they did not have any choices prior to this. See also section (ii) ‘Valorising ‘genetic connections” in this article.

8 The references given in the text ‘[6-8]’ are to scientific papers describing the experimental methods; no evidence is offered for the claim that the need for ‘this technique … is well established’.

9 It is of course possible to refer to ‘*adult* children’ in everyday speech but that qualifying adjective was also absent in these discussions, which further underlines the view that the prevailing tendency was to see the person conceived only in their infant status.

10 It is not clear precisely how these various parties were calculating their figures, given the variations between 0.1% and 0.01%, but they were all very clear that the figures were very small.

11 The ONS report that there are 52,798,300 adults in UK (ONS, http://www.ons.gov.uk/ons/rel/pop-estimate/population-estimates-for-uk--england-and-wales--scotland-and-northern-ireland/2013/sty-population-estimates.html). Using the figure given by Craven et al. of 1 in 10,000 affected individuals implies that around 5280 adults in the UK are affected. As mitochondrial diseases affect both sexes equally, this figure is in line with the estimate of 2473 affected women reported by Gorman et al. (2015).

12 The HFEA report that ‘Around 1 in 6500 children [are] thought to be born with a serious mitochondrial disorder due to faults in mitochondrial DNA’ (HFEA 2014). The Office of National Statistics report that in 2012 there were 729,674 live births in England and Wales. This suggests an incidence of 729,674/6500 = 112 affected children born per year. However, Sanderson et al. (2006) report the incidence of mitochondrial disorders as 1 in 4929. Thus, England and Wales would expect to see 729,674/4929 = 148 new cases of mitochondrial diseases in children per year.

13 The question was asked by one of the authors, KT, at the same December 2015 conference in which Walport made the remarks quoted earlier.

14 The airbrushing of the egg providers’ role also poses threats to their own health since no consideration was given in the UK debates to the health screening of potential providers nor to the handling of any incidental findings or adverse information relevant to the providers’ health arising from that screening (Applebaum et al. 2014; Haimes and Taylor 2015).

15 Tangential to the concerns about over-rapid legislation were concerns expressed about ‘slippery slopes’. Opponents of PNT/MST often raised concerns that the legalisation of these techniques would lead to other forms of genetic modification of embryos (see for example McKie 2014). These claims were contested by the proponents of PNT/MST (see for example McKie 2014, Lily Foundation 2015) who argued that altering nuclear DNA would remain illegal and that PNT/MST were solely for the purpose of removing these mitochondrial diseases from the germline of the offspring of affected women. However, soon after the House of Commons vote, the afore-mentioned researcher, Mitalipov, was quoted as saying that he hoped to be able to use the MST technique to enable older infertile women have genetically related children (Farmer 2015). Similarly, Walport’s claims, cited earlier, that approval of ‘mitochondrial donation’ provides a blueprint for future decisions on modifying the genome, is further evidence for those who fear that the slope beckons.

16 See the discussion of Mitalipov in footnote 15 above.

17 With reference to Walport’s claim that the UK legislation provides a blue print for future decisions on modifying the genome, it is sobering to note that in the world of architecture, blueprints are not the finished product that popular usage of the term implies. The technology to make blueprints always resulted in the finished product being slightly smaller than the original drawing as the medium on which the blueprint was made shrank as it dried (Liebing, 1999). Blueprints carried a warning (as do their modern, digital, equivalents) reminding architects and engineers not to take measurements directly from them, but to look elsewhere for the necessary details to construct a stable, safe and long-lasting structure.
